# Unique apicomplexan IMC sub-compartment proteins are early markers for apical polarity in the malaria parasite

**DOI:** 10.1242/bio.20136163

**Published:** 2013-09-16

**Authors:** Benoit Poulin, Eva-Maria Patzewitz, Declan Brady, Olivier Silvie, Megan H. Wright, David J. P. Ferguson, Richard J. Wall, Sarah Whipple, David S. Guttery, Edward W. Tate, Bill Wickstead, Anthony A. Holder, Rita Tewari

**Affiliations:** 1 Centre for Genetics and Genomics, School of Life Sciences, Queens Medical Centre, University of Nottingham, Nottingham NG2 7UH, UK; 2 INSERM and Université Pierre et Marie Curie, UMR_S 945 “Immunity and infection”, Centre Hospitalier Universitaire Pitié-Salpêtrière, 75013 Paris, France; 3 Institute of Chemical Biology, Department of Chemistry, Imperial College London, Exhibition Road, London SW7 2AZ, UK; 4 Nuffield Department of Clinical Laboratory Science, University of Oxford, John Radcliffe Hospital, Oxford OX3 9DU, UK; 5 Division of Parasitology, MRC National Institute for Medical Research, Mill Hill, London NW7 1AA, UK; 6 Department of Cancer Studies and Molecular Medicine, University of Leicester, Robert Kilpatrick Building, PO Box 65, Leicester Royal Infirmary, Leicester LE2 7LX, UK

**Keywords:** Polarity, ISP, *Plasmodium*

## Abstract

The phylum *Apicomplexa* comprises over 5000 intracellular protozoan parasites, including *Plasmodium* and *Toxoplasma*, that are clinically important pathogens affecting humans and livestock. Malaria parasites belonging to the genus *Plasmodium* possess a pellicle comprised of a plasmalemma and inner membrane complex (IMC), which is implicated in parasite motility and invasion. Using live cell imaging and reverse genetics in the rodent malaria model *P. berghei*, we localise two unique IMC sub-compartment proteins (ISPs) and examine their role in defining apical polarity during zygote (ookinete) development. We show that these proteins localise to the anterior apical end of the parasite where IMC organisation is initiated, and are expressed at all developmental stages, especially those that are invasive. Both ISP proteins are N-myristoylated, phosphorylated and membrane-bound. Gene disruption studies suggest that ISP1 is likely essential for parasite development, whereas ISP3 is not. However, an absence of ISP3 alters the apical localisation of ISP1 in all invasive stages including ookinetes and sporozoites, suggesting a coordinated function for these proteins in the organisation of apical polarity in the parasite.

## Introduction

The phylum *Apicomplexa* comprises over 5000 intracellular protozoan parasites, some of which are clinically important pathogens affecting humans and livestock alike. These include *Plasmodium*, the causative agent of malaria, which is responsible for approximately 1.25 million deaths annually, as well as other important parasites such as *Toxoplasma*, *Eimeria* and *Cryptosporidium* (Cavalier-Smith, 1993; Templeton et al., 2004; Wasmuth et al., 2009).

*Apicomplexa* possess a pellicle and a unique apical complex comprised of cytoskeletal structures and secretory organelles involved in parasite motility, invasion and replication (Bannister and Sherman, 2009). The pellicle is comprised of the surface plasmalemma and a double-layered inner membrane complex (IMC) located beneath the plasma membrane, which is interconnected with the cytoskeleton. The IMC is comprised of alveoli, which are flattened membrane cisternae underlying the entire plasma membrane with the exception of small gaps at the apex and base of the apicomplexan cell (Dubremetz and Torpier, 1978; Morrissette and Sibley, 2002). An actomyosin motor, located within the pellicle in the space between the plasma membrane and the IMC, drives motility and cell invasion. In *Toxoplasma gondii*, the IMC is also involved in cell division, where it serves as a scaffold for the formation of daughter cells (Nishi et al., 2008). In *Plasmodium*, formation of the IMC drives the morphological changes of the parasite during its life-cycle (Kono et al., 2012).

Proteins involved in the synthesis and organisation of the pellicle and the apical complex represent potential targets of intervention strategies to control apicomplexan diseases. Proteins known to directly associate with the IMC include those with a presumed structural function such as alveolins (IMC1a,b and h) (Khater et al., 2004; Tremp and Dessens, 2011; Tremp et al., 2008; Volkmann et al., 2012), and those associated with gliding motility including the glideosome associated proteins 40, 45 and 50 (GAP40, GAP45 and GAP50) (Ridzuan et al., 2012; Yeoman et al., 2011), which together with a myosin light chain (called MTIP - myosin A tail domain interacting protein - in *Plasmodium*) anchor the actomyosin motor complex to the IMC (Daher and Soldati-Favre, 2009). Glideosome associated proteins with multiple membrane spans (GAPMs) have also been described (Bullen et al., 2009).

Recently, four IMC sub-compartment proteins (ISPs) were described in *T. gondii* coded by genes restricted to the *Apicomplexa* (Beck et al., 2010; Fung et al., 2012). ISPs are small proteins of approximately 150 amino acids and usually characterized by a MetGly(Xaa)_2–5_CysCys sequence motif at the N-terminus (except for ISP4), but are otherwise relatively divergent and without either obvious domains, low complexity sequence or homology to other known proteins. Though a detailed analysis of the function of these proteins during asexual cell division of *T. gondii* was reported, nothing is known about their function in the malaria parasite and more importantly their role in sexual development.

Here, using the rodent malaria parasite *P. berghei* we examine the two *isp* genes in the *Plasmodium* genome, which are homologous to *T. gondii* ISP1 and ISP3. We define their expression and function during the entire life-cycle (asexual and sexual stages) involving both mammalian and vector host. We show that they have a role in the organisation of the apical end of the cell in both asexual and sexual stages. By using live cell imaging we show where the two encoded proteins are expressed during sexual stage differentiation, especially during zygote development when apical polarity is established and the apical complex is formed, resulting in the fully differentiated and invasive ookinete. We also demonstrate that these proteins are myristoylated and phosphorylated and are involved in apical membrane organisation and formation of the IMC.

## Results

### Only two paralogues of the apicomplexan ISP family are encoded in the *Plasmodium* genome

To identify ISPs in *Plasmodium*, we constructed an ISP-specific hidden Markov model from the four ISPs previously identified in *T. gondii* and used this to iteratively search the predicted proteomes of both apicomplexan and non-apicomplexan organisms. ISPs were found to be specific to *Apicomplexa*; no ISPs were identified in the predicted proteomes of 42 diverse eukaryotes from other lineages and no ISP-like sequences are present in the 27693 Transcriptome Shotgun Assembly sequences available for the free-living relative of the *Apicomplexa*, *Chromera velia* (Moore et al., 2008; Woehle et al., 2011). A similar search strategy utilizing psi-BLAST produced an identical set (data not shown).

**Fig. 1.**
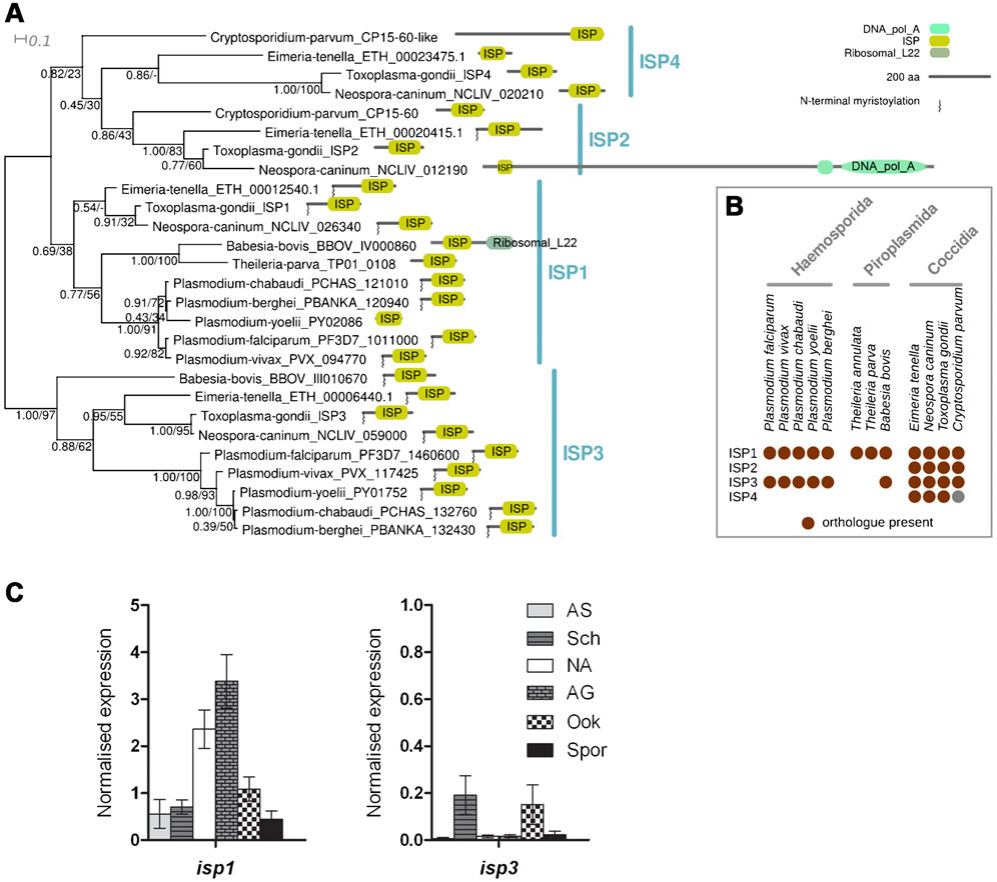
Phylogenetic analysis of ISPs in *Apicomplexa* and gene expression in wild-type *P. berghei*. A) Bayesian phylogeny of ISPs. Shown is the consensus tree produced during eight independent runs. Numbers beside nodes represent support from Bayesian posterior probabilities and maximum likelihood bootstrap values (PP/BS). A schematic representation of the predicted protein architectures (Pfam domains) is also shown. B) Distribution of ISP orthologues across apicomplexan species. C) Expression of *isp1* and *isp3* analysed by qRT-PCR, relative to *arginyl-tRNA synthetase* and *hsp70* as endogenous controls. Each point is the mean of three biological replicates each ± SEM. All asexual blood stages  =  AS; schizonts  =  Sch; non-activated gametocytes  =  NA; activated gametocytes  =  AG; ookinete  =  Ook; sporozoites  =  Spor.

The coccidian parasite genomes contained genes for four ISPs, orthologous to those in *T. gondii* (Fig. 1A,B). ISP4 sequences did not contain N-terminal glycine in a predicted myristoylation motif and also lacked the nearby conserved ‘CysCys’ motif found in most ISP1, 2 and 3 sequences, which has a predicted high probability of being S-palmitoylated (supplementary material Fig. S1). *Plasmodium* species, as well as *Babesia bovis*, contain only genes for ISP1 (PF3D7_1011000) and ISP3 (PF3D7_1460600). These families are the most conserved in terms of protein sequence and domain architecture and generally have strong predictions for N-myristoylation and palmitoylation.

### Transcripts of *isp1* and *isp3* at various developmental stages of *P. berghei* as detected by qRT-PCR

The mRNA expression profile for *isp1* and *isp3* in wild-type parasites (Fig. 1C) showed that the level of *isp1* mRNA in the blood stages was highest in gametocytes (NA and AG) before decreasing again in the mosquito stages. In contrast, *isp3* mRNA was only detected at low levels throughout the life-cycle. Enhanced expression of *isp1* mRNA in wild-type gametocytes has also been shown previously in *P. falciparum* (López-Barragán et al., 2011), and is suggestive of a requirement for ISP1 during sexual development.

### ISP1 and ISP3 show apical localisation and an IMC-like pattern during *Plasmodium* development

To determine the protein expression profile and localisation of ISP1 and ISP3, we generated a C-terminal GFP-fusion protein for both genes using the endogenous *isp1* (PBANKA_120940) and *isp3* (PBANKA_132430) and single crossover recombination (supplementary material Fig. S2A–D).

**Fig. 2.**
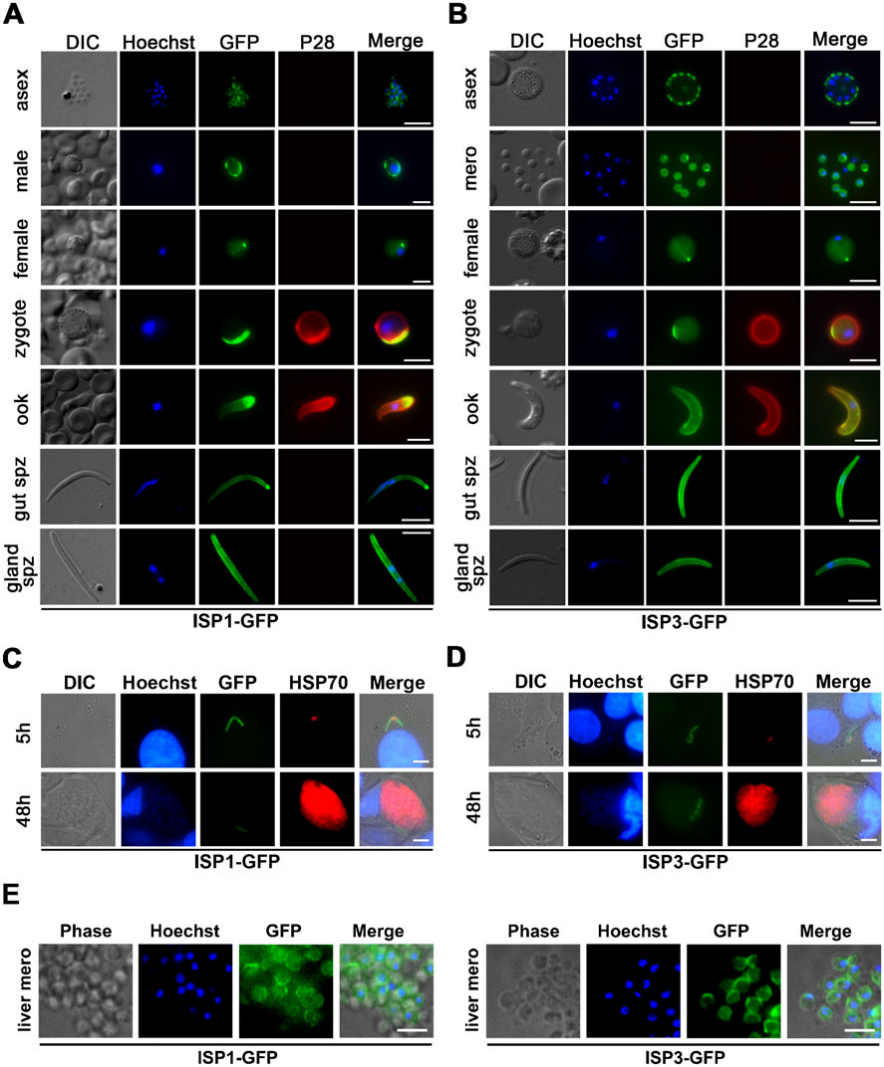
Expression of ISP1 and ISP3 during parasite development. A) Expression of ISP1-GFP in schizont (asex), activated gametocytes (male and female), zygote, ookinete (ook), and mid-gut (gut spz) and salivary gland (gland spz) sporozoites. Scale bar  =  5 µm. B) Expression of ISP3-GFP in segmenting schizont (asex), merozoites (mero), activated female gametocyte, zygote, ookinete (ook), and mid-gut (gut spz) and salivary gland (gland spz) sporozoites. For A) and B) a Cy3-conjugated antibody recognizing P28 on the surface of activated females, zygotes and ookinetes was used, nuclei were detected using Hoechst dye, and the cells were displayed by differential interference contrast (DIC). Merge is the composite of Hoechst, GFP and P28. Scale bar  =  5 µm. Expression of ISP1-GFP (C) and ISP3-GFP (D) in hepatocytic cells *in vitro* at 5 h and 48 h post invasion. An antibody to HSP70 detected parasites, hepatocyte nuclei were stained with Hoechst and the cells were displayed by differential interference contrast (DIC). Merge is the composite of Hoechst, GFP and HSP70. Scale bar  =  5 µm. E) Expression of ISP1-GFP and ISP3-GFP in liver merozoites (liver mero). Panels as in C and D. Scale bar  =  5 µm.

Despite the presence of *isp1* transcripts (Fig. 1C), the fact that *isp1* is essential for asexual blood stage development (see below) and the presence of the protein as detected by Western blotting, ISP1-GFP was only detected at low levels by fluorescence microscopy in live asexual blood stages, and by IFA (Fig. 2A). ISP1-GFP showed faint cytosolic fluorescence in activated male and female gametocytes, and in addition there was strong peripheral membrane localisation in activated male gametocytes and a strong peripheral and polarised localisation in activated female gametocytes. In zygotes, this polarised area expanded to a crescent shape and the ISP1-GFP signal was reduced in the parasite cytosol (Fig. 2A). In contrast, ISP3-GFP was clearly evident in schizonts and merozoites, while in female gametocytes and zygotes it showed a pattern similar to ISP1-GFP with both a diffuse and localised distribution (Fig. 2B). The localisation of ISP3-GFP in developing schizonts resembles an IMC-like pattern as seen for both GAP45 (Ridzuan et al., 2012) and GAP50 (Yeoman et al., 2011).

A striking difference between ISP1-GFP and ISP3-GFP was observed in ookinetes: while ISP1-GFP localisation was strong and largely limited to the apical tip of the ookinete, ISP3-GFP was distributed along the parasite periphery. Similarly, sporozoites isolated from mid-gut oocysts showed strong ISP1-GFP fluorescence at their apical tip, while ISP3-GFP was distributed throughout the cell but concentrated at the periphery of the parasite. Intriguingly, in sporozoites isolated from mosquito salivary glands, the distinctive apical localisation of ISP1-GFP was lost and instead the protein was distributed throughout the cell and concentrated at the periphery of the sporozoite, reminiscent of ISP3-GFP (Fig. 2A,B).

Next, we investigated the expression of ISP1-GFP and ISP3-GFP in liver stages (Fig. 2C,D, respectively), with both proteins showing a similar expression profile and localisation. While ISP1-GFP and ISP3-GFP fluorescence was distributed continuously along the length of extracellular sporozoites, it was disrupted at 5 h post-infection in the central protruding region of transforming intracellular sporozoites. At later stages of development (24 and 48 h), ISP1-GFP and ISP3-GFP appeared to be restricted to a small area at the parasite periphery. This distribution is reminiscent of the IMC, which is dismantled during sporozoite development in the liver (Bergman et al., 2003; Jayabalasingham et al., 2010). Interestingly, ISP1-GFP and ISP3-GFP expression increased towards the end of liver stage development, where both proteins were localised in internal structures inside the parasite (Fig. 2C,D; supplementary material Fig. S3A). Liver merozoites released in culture appeared to display a circumferential distribution of ISP1 and ISP3 (Fig. 2E).

### ISP1 and ISP3 localisation suggests a role in the organisation of apical polarity during zygote (ookinete) development and differentiation

**Fig. 3.**
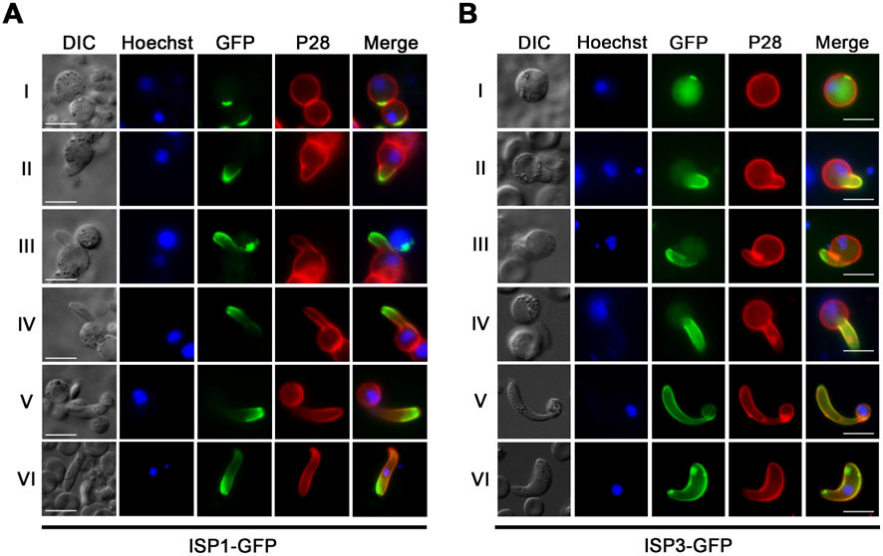
ISP1 and ISP3 define apical polarity during zygote (ookinete) development and differentiation. Localisation of ISP1-GFP (A) and ISP3-GFP (B) throughout the different stages of ookinete development (I–VI). A Cy3-conjugated antibody to P28 defines the surface of the zygote/ookinete, nuclei were detected using Hoechst dye, and the cells were displayed by differential interference contrast (DIC). Merge is the composite of Hoechst, GFP and P28. Scale bar  =  5 µm.

To further characterise the role of ISPs during *Plasmodium* ookinete development, we followed changes in the localisation of ISP1-GFP and ISP3-GFP throughout the different stages of zygote development to the fully motile and invasive ookinete (stages I–VI) (Janse et al., 1985) (Fig. 3A,B).

During stage I, both ISP1-GFP and ISP3-GFP shared a similar feature with localisation to a crescent-like structure at the periphery of the parasite, although ISP3-GFP was also distributed uniformly throughout the parasite. In stage II, a protrusion emerged from the zygote and both ISP1-GFP and ISP3-GFP were associated with this bulge. As the protrusion elongated through stages III to V, ISP1-GFP remained confined to the end of the protrusion and eventually the apical end of the mature ookinete (stage VI), whereas ISP3-GFP was spread over the entire length of the protrusion until it fully covered the emerging ookinete (Fig. 3A,B).

Whilst the IMC has been localised in the mature gametocytes of *P. falciparum* (Kono et al., 2012; Yeoman et al., 2011), its role in sexual development and differentiation has not been defined; this study shows how the IMC plays a key role in ookinete differentiation.

### Immuno-EM identifies the apical distribution of ISP1 in the ookinete

**Fig. 4.**
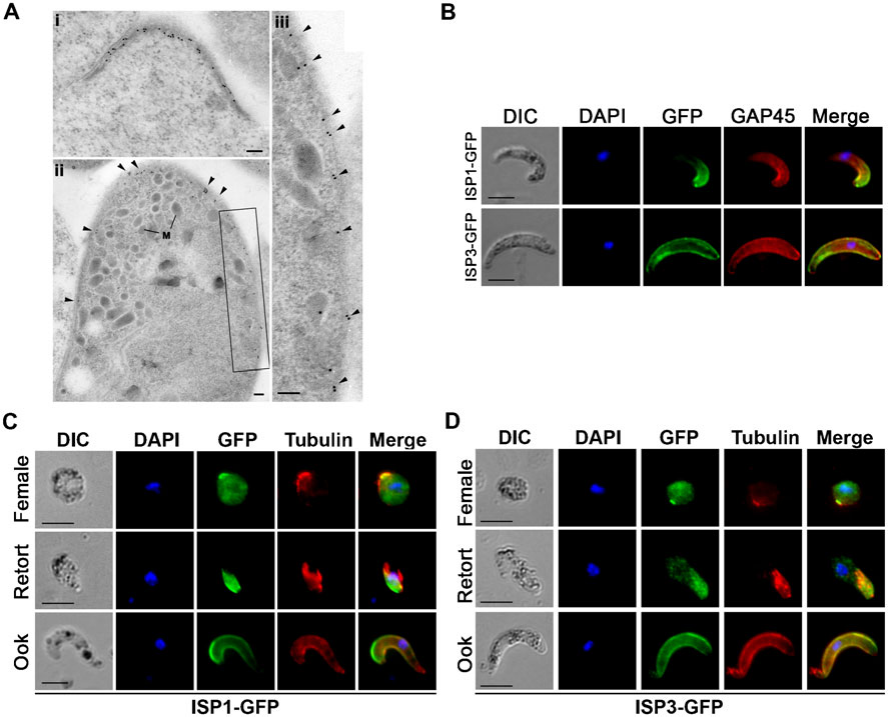
Immuno-localisation studies of ISP1 and ISP3 defines apical distribution. A) Localisation of ISP1-GFP by immuno-electron microscopy. i. part of a zygote showing a protrusion with underlying dense material labeled with gold particles. ii. Low power view of the apical end of an ookinete showing gold particles associated with the plasma membrane/IMC. Note there are more particles on one side. M – micronemes. iii. Enlargement of the enclosed area in ii showing the increased number of gold particles associated with the plasma membrane/IMC. Scale bars are 100 nm. B) Co-staining of ISP1-GFP and ISP3-GFP expressing ookinetes with antibodies to GFP (green) and IMC marker GAP45 (Red). C–D) Indirect immunofluorescence showing the co-localisation of ISP1-GFP and ISP3-GFP with respect to the cytoskeletal marker α-tubulin, during gametocyte and ookinete stages (Scale bar  =  5 µm). Strong co-localisation can be seen at the apical end for both ISP1 and ISP3, with α-tubulin (red). Nuclei were detected using Hoechst dye, and the cells were displayed by differential interference contrast (DIC). Merge is the composite of Hoechst, GFP and GAP45/tubulin. Scale bar  =  5 µm.

To determine whether ISP1-GFP is associated with the plasma membrane or the underlying IMC, we performed immuno-EM using a mouse anti-GFP antibody and an anti-mouse Ig antibody conjugated with colloidal gold particles. In zygote development, electron-dense structures protruding from the early ookinetes (stage I) were strongly labelled substantiating the polar pattern of GFP expression observed in light microscopy (Fig. 4Ai). In the case of fully formed ookinetes, labelling was observed along the IMC complex specifically at the apical end, although a few gold particles were seen associated with the apical collar and ring structures (Fig. 4Aii,iii). It was repeatedly observed that there were more gold particles labelling the pellicle along one side of the parasite than the other (Fig. 4Aiii).

### ISP1 and ISP3 show partial apical co-localisation with IMC marker GAP45 and α-tubulin

To investigate the expression pattern of ISP1 and ISP3 in greater detail with respect to the organisation of apical polarity, we performed co-localisation studies for ISP1 and ISP3 with the IMC marker GAP45 and the cytoskeletal marker α-tubulin in gametocytes and ookinetes (Fig. 4B–D; supplementary material Fig. S3B,C). Tomography studies suggest a possible association of microtubules and the IMC at the apical end of sporozoites (Kudryashev et al., 2010) but little is known for ookinete stages. Both markers showed partial co-localisation with the ISPs at the apical end suggesting a similar association during sexual stages. Co-localisation of α-tubulin with ISP1 and ISP3 in female gametocytes substantiates their apical polarity as observed earlier during zygote development (Fig. 4C,D; supplementary material Fig. S3B,C).

### Differential subcellular localisation of ISP1 and ISP3

**Fig. 5.**
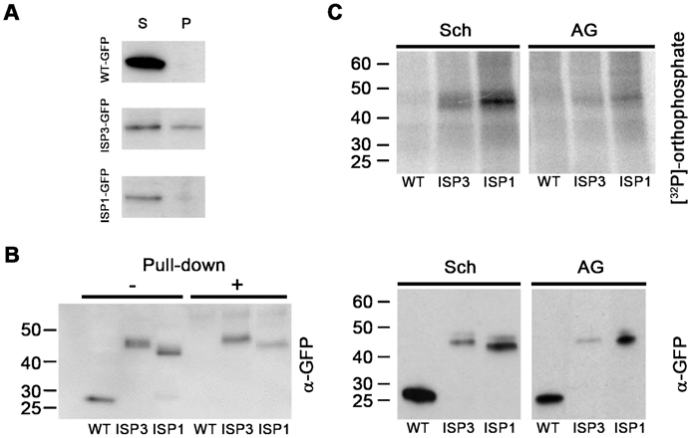
Biochemical characterisation of ISP1 and ISP3. A) Detection of untagged GFP, ISP1-GFP and ISP3-GFP in NP40-soluble (S) and NP40-insoluble particulate (P) subcellular fractions of blood stage parasites (schizont) by western blotting with anti-GFP antibodies. B) Myristoylation of ISP1-GFP and ISP3-GFP detected using YnMyr labelling and click chemistry. Untagged GFP-, ISP1-GFP- and ISP3-GFP-expressing parasites were labelled and lysates were run directly (−) or following affinity purification (+) on an SDS-PAGE gel and protein detected with anti-GFP antibodies. The sizes of molecular mass markers are shown to the left. C) *In vivo* phosphorylation of ISPs. Upper panel: autoradiograph showing [^32^P]-phosphorylation of immunoprecipitated GFP-proteins from schizont (Sch) and activated gametocyte (AG) lysates of WT-GFP, ISP3-GFP and ISP1-GFP parasites, resolved on SDS-PAGE. Lower panel: corresponding Western blot using anti-GFP antibody on the fractionated immunoprecipitates. Molecular mass markers are shown to the left.

Since ISP1-GFP and ISP3-GFP show an IMC-like pattern and are partially co-localised with α-tubulin and GAP45, we performed subcellular fractionation experiments using NP-40 solubilisation to determine whether or not ISP1-GFP and ISP3-GFP were associated with the detergent-resistant cytoskeletal meshwork of the IMC. While ISP1-GFP was found exclusively in the soluble fraction (NP-40 detergent solubilized), ISP3-GFP was associated both with the soluble and particulate (NP-40 insoluble) fractions in schizonts (Fig. 5A), showing that ISP3-GFP might be partially embedded in the detergent-resistant meshwork associated with IMC membranes, whereas ISP1 is not. These results suggest different properties for ISP3 compared to the protein in *T. gondii*, where ISP1-4 were all completely solubilised after NP-40 detergent extraction (Beck et al., 2010; Fung et al., 2012) and located to a specific sub-compartment of the IMC distinct from the protein meshwork underlying the IMC membranes.

### ISP1-GFP and ISP3-GFP are myristoylated proteins

To test whether ISP1 and ISP3 are myristoylated in live *P. berghei*, we treated ISP1-GFP and ISP3-GFP expressing cells with the alkyne-tagged myristic-acid analogue YnMyr. This probe can be utilized by *N*-myristoyltransferase (NMT) in place of myristic acid and co-translationally transferred to the N-terminal glycine residue of susceptible proteins (Heal et al., 2012). Bio-orthogonal ligation or “click” chemistry was then used to label all proteins that had incorporated the YnMyr probe with a biotinylated capture reagent, which is ligated to the alkyne tag, allowing the selective affinity pull-down of tagged proteins with streptavidin-conjugated resin (Heal et al., 2012). The resulting purified myristome was analysed by Western blot using anti-GFP antibody and showed the presence of ISP1-GFP and ISP3-GFP amongst the myristoylated proteins (Fig. 5B). Untagged GFP was used as a control and was not enriched, as expected.

### ISP1 and ISP3 are phosphorylated in both asexual (schizont) and sexual stages (activated gametocytes)

It has been suggested that protein phosphorylation plays a role in the organisation of the molecular machinery required for gliding motility and invasion (Ridzuan et al., 2012; Thomas et al., 2012); therefore, we investigated whether ISP1-GFP and ISP3-GFP were phosphorylated *in vivo*. ISP1- and ISP3-GFP parasite lines were metabolically labelled for 30 min with [^32^P]-orthophosphate, lysed and then ISP1-GFP and ISP3-GFP immunoprecipitated to assess their phosphorylation status in schizonts and activated gametocytes. We found that both ISP1-GFP and ISP3-GFP were phosphorylated in both stages (Fig. 5C, upper panel). Interestingly, data from only one of the previous global phosphoproteomics studies (Treeck et al., 2011) suggested that ISP3 (but not ISP1) was phosphorylated in *P. falciparum* schizonts, whereas the other two studies did not detect ISP phosphorylation (Lasonder et al., 2012; Solyakov et al., 2011).

### ISP1 has a likely essential function for parasite development, but ISP3 is dispensable

To elucidate the function of ISP1 and ISP3, we attempted to delete both *isp1* and *isp3* using a double homologous recombination strategy, in which each gene would be replaced with a *T. gondii dhfr/ts* selectable marker cassette (supplementary material Fig. S2E–H).

**Fig. 6.**
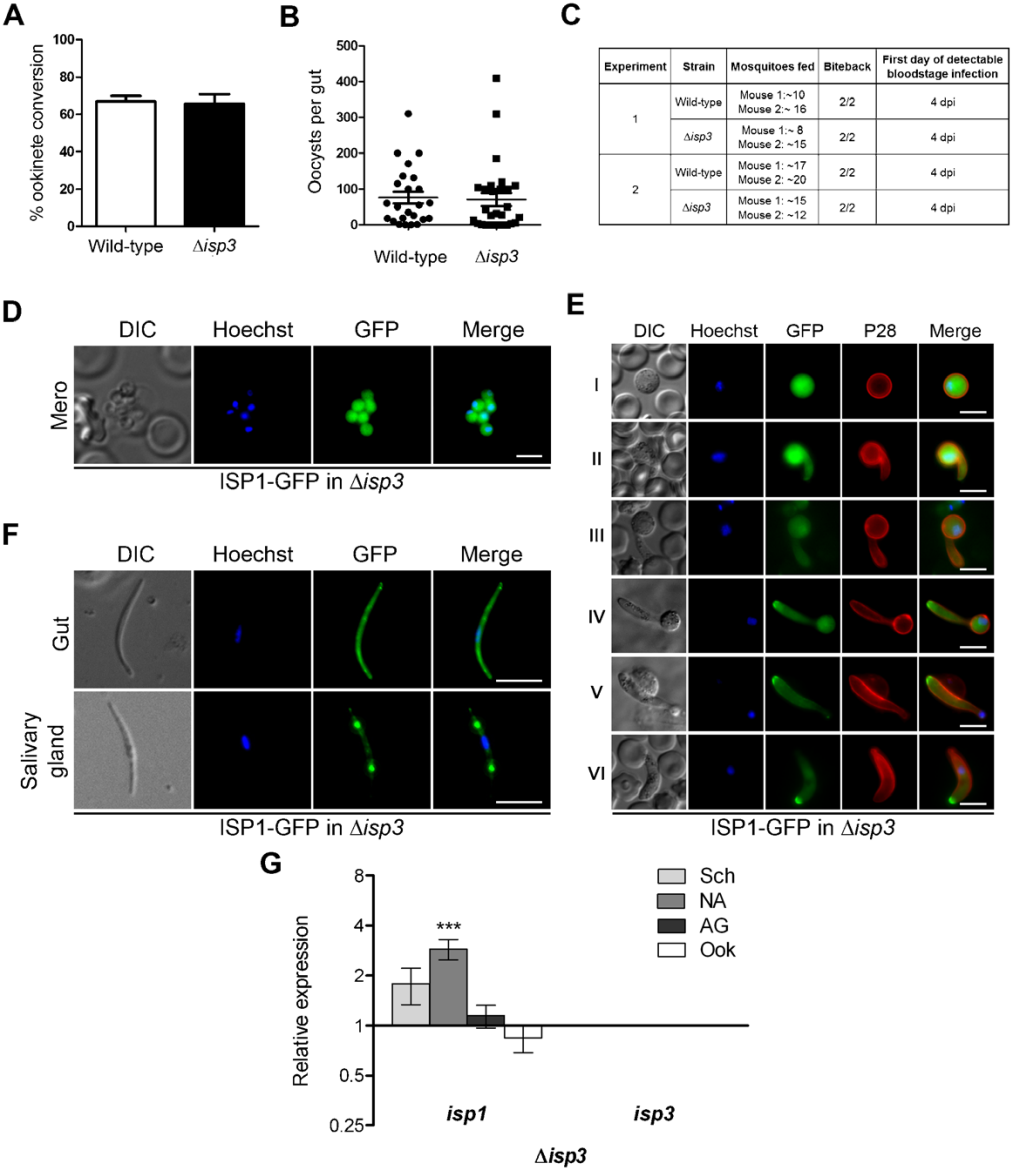
Effect of ISP3 deletion on the parasite life cycle and on the localisation of ISP1. A) Ookinete conversion in wild-type and Δ*isp3* parasites (error bar  =  ±SD; *n* = 3). B) Oocysts per mosquito gut (day 14 post-infection; bar  =  arithmetic mean; *n = 3*) of wild-type or Δ*isp3* parasite-infected mosquitoes from three independent experiments. Overall infection rate was 90% for both wild-type and Δ*isp3* parasites. C) Bite-back experiments indicating successful transmission of both wild type and Δ*isp3* parasites from mosquito to mouse. Infected mosquitoes were allowed to feed and the mice were monitored for blood stage parasitaemia (days post infection, dpi) indicative of successful liver and blood stage infection. D) Altered localisation of ISP1 in Δ*isp3* merozoites. Scale bar  =  5 µm. E) ISP1-GFP localisation during each stage of zygote differentiation to ookinete in Δ*isp3* line. Stage of development (I–VI) is indicated to the left of each row. A Cy3-conjugated antibody to P28 defines the surface of the zygote/ookinete, nuclei were detected using Hoechst dye, and the cells were displayed by differential interference contrast (DIC). Merge is the composite of Hoechst, GFP and P28. Scale bar  =  5 µm. F) Localisation of ISP1-GFP in Δ*isp3* gut and salivary gland sporozoites. Scale bar  =  5 µm. G) Relative expression of *isp1* and *isp3* in Δ*isp3* parasites compared to wild-type controls using the ΔΔC_t_ method. The level of *isp1* mRNA is significantly increased in non-activated gametocytes (*P*<0.001). Bars represent the mean of 3 biological replicates ± SEM. Schizonts  =  Sch; non-activated gametocytes  =  NA; activated gametocytes  =  AG; ookinetes  =  Ook. ***  =  *P*<0.001.

We transfected wild-type parasites with the *isp1* knockout construct on seven independent occasions but were unable to generate a transgenic line, indicating that this gene is likely essential during asexual development. For *isp3*, deletion mutants (Δ*isp3*) were obtained and we analysed two clones from two independent transfections. Both Δ*isp3* clones developed normally in the asexual blood stages, formed gametocytes, which exflagellated and converted to ookinetes normally, infected mosquitoes in numbers comparable to the wild-type, were transmitted to naïve mice in bite back experiments, and completed the full life-cycle (Fig. 6A–C).

### Apical polarity and localisation of ISP1 is altered in the absence of ISP3 suggesting coordinated function

We examined ISP1-GFP expression and localisation in the Δ*isp3* parasite to investigate whether the apical expression profile of ISP1-GFP is affected by deletion of *isp3*. For this we generated a C-terminal GFP fusion protein for ISP1 using the Δ*isp3* parasite, by single crossover recombination and selection with WR99210 (supplementary material Fig. S2I,J).

Interestingly, we observed altered localisation of ISP1-GFP in schizonts and merozoites in the *isp3* mutant cells (Fig. 6D). In female gametocytes and zygotes, ISP1-GFP fluorescence was no longer localised to the periphery (Fig. 6E, stage I). Instead, the signal was distributed throughout the cytosol. We then followed the developmental changes during zygote differentiation to ookinetes and found that only after 10–12 hours when the cells had progressed to stage IV (Janse et al., 1985) did the GFP signal begin to localise at the apical tip of the converting cell. However, the pattern in mature ookinetes remained different from that observed for ISP1-GFP in the wild-type background since the signal in the Δ*isp3* background was reduced and more restricted to the apical end (Fig. 6E). In both gut- and salivary gland-derived sporozoites, again the distribution of ISP1-GFP in Δ*isp3* parasites differed in its apical and membrane distribution (Fig. 6F) from that observed in the wild-type (Fig. 2A).

To further characterise Δ*isp3* parasites and to investigate a possible role for *isp1* in compensating for the loss of *isp3*, we performed qRT-PCR analysis of the expression of *isp1* in Δ*isp3* lines (Fig. 6G). In Δ*isp3* parasites, *isp1* mRNA was significantly increased in non-activated gametocytes (*P*<0.001). Increases were identified at other stages of the parasite life-cycle although they did not reach statistical significance.

## Discussion

Apicomplexan parasites including the genera *Plasmodium*, *Toxoplasma*, *Babesia*, *Cryptosporidium*, *Theileria* and *Eimeria* are major causes of disease in humans and animals. The unifying feature of these parasites is an apical end comprised of a surface plasmalemma and an inner membrane complex (IMC). These structures are involved in parasite motility and host cell invasion and are hence an important area to target for intervention. The identification of ISP proteins opens new avenues to understand the role of sub compartment IMC proteins in parasite invasion and also their role during apical end formation and polarity, for which very little is known.

The distribution and phylogeny of ISPs suggest duplication of the *isp1* gene in the ancestor of the *Coccidia* (which build complete conoids at the apical end of the parasite), giving rise to *isp2* and *isp4*. Interestingly, *Theileria* species have lost the *isp3* gene, such that they now encode only ISP1. Invasion mechanisms in *Theileria* are different from those of other *Apicomplexa* and these species use host factors during development (Shaw, 2003), suggesting the evolution of ISPs could be related to parasite invasion strategies.

Following on from this, we show that of the two ISPs identified in *Plasmodium*, only ISP1 is likely essential for parasite development. It is possible that the function of ISP1 can be dissected in future studies using either the FLP/FRT (Lacroix et al., 2011) or recombinant Cre (Collins et al., 2013) systems of conditional mutagenesis or by promoter swap with a schizont specific promoter as has been demonstrated for MyoA and CDPK1 in recent studies (Sebastian et al., 2012; Siden-Kiamos et al., 2011). In addition some of the approaches described in PlasmoGEM can be used to determine whether longer flanking regions for recombination would facilitate ISP1 gene disruption (Pfander et al., 2013). In contrast, all the ISPs in *T. gondii* can be deleted individually and a functional redundancy between the ISPs has been suggested. Only *Tgisp2* null mutants cause a growth defect in *T. gondii* and exhibited changes to their mode of replication, whereas null mutants for *isp1*, *isp3* and *isp4* showed no major changes in fitness (Beck et al., 2010; Fung et al., 2012). It is possible that the reduction of *isp*s from four to two genes in *Plasmodium* has decreased functional redundancy between ISP1 and ISP3, making *isp1* essential in *P. berghei*.

The distribution of ISP3 around the periphery of merozoites, ookinetes and sporozoites we observed in this study of *P. berghei* differs substantially from its location in *T. gondii*. In *T. gondii* tachyzoites ISPs have a more compartmentalised distribution: ISP1 at the apical cap of the parasite, ISP2 and ISP4 at the centre, and ISP3 throughout the central area and the basal end. In *T. gondii*, ISPs display a more hierarchical organisation whereas in *Plasmodium* such an organisation is not evident. For example, in *T. gondii* the presence of ISP1 at the apical cap excludes the other ISPs from this region (Beck et al., 2010; Fung et al., 2012), which is not seen in *P. berghei*. Only ISP1 is present in the anterior region and there is a clear boundary at the front and back, delineated by the presence of centrin-2. In contrast, in *Plasmodium* the two ISPs show a different expression profile, with no hierarchy in their organisation, less discrete boundaries, and both show apical polarity, although ISP1 is more restricted to the apical end in ookinetes. Some of the protein appears to be soluble and not associated with the plasma membrane. It is unknown whether this is due to a fraction that has not been N-myristoylated, although clearly at least some of ISP1 and ISP3 is myristoylated as shown in this study. Whilst myristoylation alone may be insufficient to bind ISPs to the IMC, nevertheless it is likely to make an important contribution. We are currently developing N-myristoylation transferase inhibitors, which will be useful to test this hypothesis (Yu et al., 2012).

The mechanism by which ISP1-GFP is restricted to the apical end of *Plasmodium* parasites is unknown. This apical polarity also shows dynamic changes as seen during development in the mosquito mid-gut through to salivary gland sporozoites. ISP1 is present in the *P. falciparum* blood stage palmitome (Jones et al., 2012) and whereas N-myristoylation is irreversible, palmitoylation is not. The apparent redistribution of ISPs during parasite development may result from either degradation of pre-existing ISPs in one location and deposition of newly synthesised protein in another position, flow of acylated protein within the membrane phase or the reversible palmitoylation of ISPs. For *T. gondii*, Beck et al. suggest a model where localisation of ISP1 to the IMC is dependent on a kinetic trapping mechanism (Beck et al., 2010; Resh, 2006) involving myristoylation and subsequent palmitoylation (Beck et al., 2010; Fung et al., 2012). Whilst a similar model may explain the targeting of ISP1 to the apical end in *Plasmodium*, the dynamic state of the ISPs suggests that kinetic trapping alone cannot fully explain their localisation, coordinated function and redistribution. Moreover, since both ISPs in *Plasmodium* are localised to the apical end but exist in a dynamic state, the mechanism behind this may differ between *Toxoplasma* and *Plasmodium*. The redistribution of ISP1 in the absence of ISP3 suggests that these proteins act in a coordinated fashion and they may both interact with other proteins that control their apical localisation, which remains to be investigated.

The other potentially reversible modification of ISPs is phosphorylation through the action of protein kinases and phosphatases, although the relevant enzymes have not yet been identified. Phosphorylation has been shown to be important for the glideosome machinery (Green et al., 2008; Ridzuan et al., 2012), and phosphoproteomic studies have identified one phosphorylation site on *P. falciparum* ISP3 (Treeck et al., 2011). In *T. gondii* ISP2 and ISP3, but not ISP1 and ISP4 are present in the tachyzoite phosphoproteome (Treeck et al., 2011). Such modifications may also affect the insertion of the proteins into the membrane or their interactions with other proteins.

Several components of the motor complex, in particular proteins associated with the IMC and involved in gliding motility such as MTIP and GAP45 are also regulated by phosphorylation. For instance, MTIP binding to myosin A (MyoA) is phosphorylation-dependent and GAP45 is multiply phosphorylated *in vivo* and phosphorylated by calcium-dependent protein kinase 1 (CDPK1) and protein kinase B (PKB) *in vitro* (Douse et al., 2012; Ridzuan et al., 2012; Thomas et al., 2012). The phosphorylation-dependent regulation of these motor complex proteins suggest that in addition to myristoylation, phosphorylation could also be important in the regulation of ISP1 and ISP3 function in *P. berghei*.

We have shown in this study that *isp1* mRNA is maximally expressed in gametocytes, just prior to expression of the protein in ookinetes, supporting the hypothesis that ISP1 can compensate for the absence of ISP3 but the distribution of the protein is affected. This indicates a coordinated function of ISP1 and ISP3 in their expression. In contrast to *Toxoplasma*, where ISP1 has been suggested to have a gatekeeping effect by preventing access of ISP3 to the apical end (Beck et al., 2010) we observed that ISP3 is required for proper apical localisation of ISP1. ISP1 does not need to localise to the membrane or IMC of zygotes for the protrusion of the differentiating ookinete to form in stage I; at this stage, it is a marker for where the apical end begins to form. In summary our observations indicate that while only ISP1 is likely to be essential for malaria parasite development; both ISPs are involved in apical end polarity during sexual and asexual development.

## Materials and Methods

### Ethics statement

All animal work has passed an ethical review process and was approved by the United Kingdom Home Office. Work was carried out in accordance with the United Kingdom ‘Animals (Scientific Procedures) Act 1986’ and in compliance with ‘European Directive 86/609/EEC’ for the protection of animals used for experimental purposes. The permit number for the project licence is 40/3344.

### Animals

Tuck's Original (TO) (Harlan) outbred mice were used for all experiments.

### Bioinformatic analysis

To identify ISP-like proteins, an iterative search was performed using HMMER3 (Eddy, 2009). A specific hidden Markov model was made from an alignment of *T. gondii* ISP1, 2, 3 and 4, and used to search the predicted proteomes of 12 apicomplexan parasites (EuPathDB; http://eupathdb.org) and a further 42 eukaryotes for which complete genome sequence is available (see Wickstead et al., 2010 for full list). Identified ISP proteins (e-value <0.001) were incorporated into a new hidden Markov model and the search performed without identifying additional sequences.

Identified ISP sequences were aligned using MAFFT6.24 (Katoh et al., 2002) and well-aligned blocks were used to infer a Bayesian phylogeny using the metropolis-coupled Markov chain Monte Carlo method as implemented by MrBayes3.2 (Ronquist and Huelsenbeck, 2003). Eight independent runs of 200,000 generations were performed from random start trees (initial 40,000 generations discarded) using the WAG substitution matrix with a gamma-distributed variation in substitution rate approximated to 4 discrete categories (shape parameter estimated from the data). Support for the inferred phylogeny was also provided from a majority-rule consensus of 100 bootstrap replicate maximum likelihood trees inferred with RAxML 7.2.8 (Stamatakis, 2006), (eight gamma categories, LG matrix, other parameters at default).

Protein domain architectures were predicted from the models in Pfam26 with e-value <0.001 and the inclusion of the custom-build ISP domain. Possible myristoylation was predicted using N-Myristoyltransferase (NMT) predictor (Eisenhaber et al., 2003). S-palmitoylation was predicted using CSS-Palm 3.0 (Xue et al., 2006); http://csspalm.biocuckoo.org.

### Generation of targeting constructs

To tag both ISP1 and ISP3 by single homologous recombination at the C-terminus with GFP, the p277 vector containing a human *dhfr* selection cassette was used (supplementary material Fig. S2A). A 672 bp region of *isp1* starting 331 bp downstream of the ATG start codon and omitting the stop codon was amplified using the primers T1281 (5′-CCCCGGTACCCGACTAAAAAATAACATCCAAATAGTTG-3′) and T1282 (5′-CCCCGGGCCCATTTTTTTTATAATCTCTCATAATATAAATAAATAA-3′) and inserted into the vector using *Kpn*I and *Apa*I restriction sites. For *isp3*, a 769 bp region of the gene locus was amplified starting 319 bp upstream of the ATG start codon and omitting the stop codon using the primers T0641 (5′-CCCCGGTACCCCTATGATTATAACGAAAAATAAAACCCC-3′) and T0642 (5′-CCCCGGGCCCAGCAGTTAAGCAATGCTTTTTTATAAATTC-3′) and inserted into p277 using *Kpn*I and *Apa*I restriction sites. Before transfection, the *isp1* tagging construct was linearised using *Cla*I and the *isp3* tagging construct was linearised using *Bcl*I.

The targeting vectors for *isp1* and *isp3* deletion by double homologous recombination were constructed using the pBS-DHFR plasmid, which contains a *T. gondii* dhfr/ts expression cassette conveying resistance to pyrimethamine flanked by polylinker sites (supplementary material Fig. S2E). For both genes, 5′ fragments upstream of the respective gene were inserted into *Apa*I and *Hind*III restrictions sites upstream of the dhfr/ts cassette and fragments of the 3′ downstream end were cloned into *Eco*RI and *Xba*I restriction sites downstream of the dhfr/ts cassette. The linear targeting sequence was released using *Apa*I/*Xba*I. For *isp1* PCR primers were N0561 (5′-CCCCGGGCCCGTAAATTAAAGATCAAATTAGAGAAG-3′) and N0562 (5′-GGGGAAGCTTGATATAAATATACATACACACATAC-3′) to amplify the 437 bp 5′ fragment and N0563 (5′-CCCCGAATTCGGAGAAGAACAATTAAAAAGAGTAG-3′) and N0564 (5′-GGGGTCTAGAGGTGAATGAAATAAGATCATGGGGG-3′) to amplify the 439 bp 3′ fragment. For *isp3* PCR primers were N0361 (5′-CCCCGGGCCCGGCTATTTTTGCATATAAATTCTGAATTG-3′) and N0362 (5′-GGGGAAGCTTGCATATATATCTATATTGGATTTGTTG-3′) to amplify the 500 bp 5′ fragment and N0363 (5′-CCCCGAATTCGCTATTGCCTTTGCAATGGATAGTC-3′) and N0364 (5′-GGGGTCTAGACAAATAATGTTTATCTAATATATTGGG-3′) to amplify the 463 bp 3′ fragment.

The *P. berghei* ANKA line 2.34 was then transfected by electroporation (Janse et al., 2006). Electroporated parasites were immediately mixed with 150 µl of reticulocyte rich blood, incubated at 37°C for 30 min and injected intraperitoneally (i.p.) into naïve mice. Pyrimethamine (Sigma) at 7 mg/ml was supplied in the drinking water for four days or WR99210 for the generation of ISP1-GFP in Δ*isp3* (gift from Jacobus Pharmaceuticals) was given by i.p. injection from day 1 post infection. Mice were monitored for 15 days for emergence of parasites and drug selection was repeated after passage to a second mouse. Resistant parasites were then used for genotypic analysis and limiting dilution cloning.

### Genotypic analyses of parasites

For the genotypic analyses of C-fusion GFP tagged parasites, a diagnostic PCR reaction was performed as outlined in supplementary material Fig. S2B,C,I. Primer 1 (For *isp1* intT128 5′-CTATACACAGCATATATCTATATACATTGGC-3′; for *isp3* intT064 5′-CAATAAATTACATACATTGTTTAAGTCC-3′) and primer (ol492, 5′-ACGCTGAACTTGTGGCCG-3′) were used to determine correct integration of the *gfp* sequence at the targeted locus.

Two diagnostic PCRs were performed for *isp3* gene knockout parasites as outlined in supplementary material Fig. S2F,I. Primer 3 (intN36 5′-GTACATTATTTTTGATTCGTTCATACTATAG-3′) and Primer 4 (ol248, 5′-GATGTGTTATGTGATTAATTCATACAC-3′) were used to determine successful integration of the targeting construct at the respective gene locus. Primers 5 (N36ko1 5′-CAATTAGAGTAGCATTTCCTGATGG-3′) and 6 (N36ko2 5′-GACTATCCATTGCAAAGGCAATAGC-3′) were used to verify deletion of the gene.

For Southern blotting, genomic DNA from wild type and mutant parasites was digested with *Xba*I and *Spe*I and separated on a 0.8% agarose gel before blotting onto a nylon membrane (GE Healthcare). A probe was generated from a PCR fragment homologous to the 5′ region just outside of the targeted region using the AlkPhos direct labelling kit (GE Healthcare) according to manufacturer's instructions (supplementary material Fig. S2G,J).

For pulsed field gel electrophoresis (PFGE) chromosomes of wild type and mutant parasites were separated on a CHEF DR III (BioRad) system using a linear ramp of 60–500 s for 72 h at 4 v/cm. The gel was blotted onto a nylon membrane and hybridized with a probe recognizing both the resistance cassette in the targeting vector and the 3′ UTR of the *P. berghei dhfr*/*ts* locus on chromosome 7 (supplementary material Fig. S2H).

### Phenotypic analyses

To initiate infections, blood containing 5×10^6^ parasites was injected i.p. into mice. Asexual stages and gametocyte production were monitored on Giemsa stained thin smears. 4–5 days post infection, exflagellation and ookinete conversion were examined as described previously (Guttery et al., 2012) with a Zeiss AxioImager M2 microscope (Carl Zeiss, Inc) fitted with an AxioCam ICc1 digital camera.

To analyse mosquito transmission, 30–50 *Anopheles stephensi* SD 500 mosquitoes were allowed to feed for 20 min on anaesthetized infected mice whose asexual parasitaemia had reached ∼7% and were carrying comparable numbers of gametocytes as determined on Giemsa stained blood films.

To assess mid-gut infection, approximately 20 guts were dissected from mosquitoes on day 14 post feeding and mounted under Vaseline-rimmed cover slips after staining with Hoechst 33342 for 10–15 min. Oocyst were counted on an AxioCam ICc1 digital camera fitted to a Zeiss AxioImager M2 microscope using a 63× oil immersion objective. On day 21 post-feeding another 20 mosquitoes were dissected and their guts and salivary glands crushed separately in a loosely fitting homogenizer to release sporozoites, which were then quantified using a haemocytometer or used for imaging. Mosquito bite back experiments were performed 21 day post feeding using naïve mice and blood smears were examined.

### Purification of schizonts, gametocytes and ookinetes

Blood cells obtained from infected mice (day 5 post infection) were placed in culture for 24 h at 37°C (with rotation at 100 rpm) and schizonts were purified the following day on a 60% v/v NycoDenz (in PBS) gradient, harvested from the interface and washed. (NycoDenz stock solution: 27.6% w/v NycoDenz in 5 mM Tris-HCl, pH 7.20, 3 mM KCl, 0.3 mM EDTA).

Purification of gametocytes was achieved using a protocol modified from (Beetsma et al., 1998). Mice were treated i.p. with 0.2 ml of phenylhydrazine (Sigma, 6 mg/ml) in PBS to encourage reticulocyte formation four days prior to infection. On day four post-infection mice were treated with sulfadiazine (Sigma, 20 mg/l) in their drinking water for three days to eliminate asexual blood stage parasites. On day six post-infection mice were bled by cardiac puncture into heparin and gametocytes separated from uninfected erythrocytes on a 48% (v/v) NycoDenz in coelenterazine loading buffer (CLB; PBS, 20 mM HEPES, 20 mM Glucose, 4 mM sodium bicarbonate, 1 mM EGTA, 0.1% w/v bovine serum albumin, pH 7.25) gradient. Gametocytes were then harvested from the interface and washed twice in RPMI 1640 before activation of gamete formation in ookinete medium.

Blood cells from day 5 post infection mice were placed in culture for 24 hrs at 20°C for ookinete production as described above. Ookinetes were purified on a 63% NycoDenz gradient (v/v in CLB), harvested from the interface, washed and used for downstream experiments.

### Subcellular fractionation

Schizonts obtained from untagged GFP, ISP1-GFP and ISP3-GFP parasite lines were prepared as described above and subcellular fractionation was performed using a method adapted from (Beck et al., 2010). Parasite pellets were resuspended in PBS containing protease inhibitors (Roche) and 0.5% NP40, lysed for 20 min at 4°C and centrifuged for 15 min at 16,000 g at 4°C. Equivalent amounts of the supernatant (soluble fraction) and of the washed pellet resuspended in Laemmli buffer (particulate fraction) were then subjected to SDS-PAGE and analysed by Western blot with anti-GFP rabbit polyclonal antibody (Invitrogen, 1:1,250 dilution) using the Western Breeze Chemiluminescent Anti-Rabbit kit (Invitrogen) according to the manufacturer's instructions.

### ISP *in vivo* phosphorylation

As described previously (Guttery et al., 2012), schizonts and activated gametocytes (purified as described above) were washed in phosphate-free Krebs buffer and metabolically labelled with 3–5 MBq [^32^P]-orthophosphate (Perkin Elmer) in the same buffer for 30 min at 20°C or 37°C for activated gametocytes and schizonts, respectively. Following two washes in phosphate-free Krebs buffer, the labelled parasites were lysed for 30 min at 4°C in lysis buffer (10 mM Tris-HCl pH 7.5, 150 mM NaCl, 0.5 mM EDTA, 0.5% NP-40) supplemented with protease and phosphatase inhibitors (both Roche). The resulting lysate was centrifuged at 20,000 g for 10 min and the supernatant collected. GFP tagged proteins were then isolated using GFP-TRAP beads (ChromoTek) according to the manufacturer's instructions and the immunoprecipitated proteins were subsequently resuspended in Laemmli sample buffer for separation by SDS-PAGE. [^32^P]-labelled proteins were visualized using a phosphorimager (Molecular Dynamics) and GFP-tagged proteins analysed by Western blot as described above.

### Metabolic labelling and purification of *N*-myristoylated proteins

To metabolically label *N*-myristoylated proteins, the blood of one infected mouse was placed in schizont medium containing 50 µM YnMyr (tetradec-13-ynoic acid) and grown overnight at 37°C before purification as described above.

Parasite proteins were extracted using 1% Triton X-100 in 10 mM Na_2_PO_4_, pH 8.2 with protease inhibitors (EDTA-free, Roche) incubated overnight at 4°C with rotation. Extracts were pelleted and the concentration of protein in the supernatant determined by DC protein assay (Bio-Rad).

Protein lysates were adjusted to 1 mg/ml with lysis buffer; premixed labelling reagents (100 µM AzTB (azido-TAMRA-biotin), 1 mM CuSO_4_, 1 mM TCEP, 100 µM TBTA, final concentrations) were added as described previously (Heal et al., 2012) and samples vortexed for 1 h RT, then quenched by the addition of 10 mM EDTA, followed by 10 volumes of ice-cold MeOH. Samples were left at −80°C overnight and then proteins pelleted by centrifugation at 17,000 ×g for 20 min at 4°C. Pellets were washed with ice-cold MeOH, then air-dried for ∼15 min.

Protein was resuspended at 10 mg/mL in 2% SDS, 10 mM EDTA in PBS, and then diluted to 1 mg/mL with PBS. Aliquots were removed, mixed with 4× sample loading buffer (NuPAGE LDS sample buffer) and 2-mercaptoethanol (4% final), then heated for 3 min at 95°C for pre-enrichment analysis. DTT (from a fresh 100× stock in water) was added to the remaining solution to give a final concentration of 1 mM. Proteins were incubated with Dynabeads® MyOne^TM^ Streptavidin C1 (pre-washed 3× 0.2% SDS in PBS) for 2 h at RT with rotation. Following removal of the supernatant, beads were washed 3× with 1% SDS in PBS, then boiled for 10 min in sample loading buffer to elute bound proteins. Aliquots of supernatant were also taken for analysis.

For immunoblotting, proteins were transferred to PVDF membranes, membranes were blocked (5% dried skimmed milk in TBS 0.1% Tween-20), then probed with anti-GFP (rabbit polyclonal, 1:2,000, Invitrogen), followed by anti-rabbit HRP secondary antibody (goat anti-rabbit, 1:10,000, Invitrogen) in blocking solution, and developed with Luminata Crescendo Western HRP substrate (Millipore) according to the manufacturer's instructions and on a Fujifilm LAS 3000 imager.

### Immuno electron microscopy

Zygotes and ookinetes were fixed in 2% paraformaldehyde in 0.1M phosphate buffer, dehydrated and embedded in LR White acrylic resin. For immune-staining, thin sections on nickel/formvar grids were floated on drops of 1% BSA in PBS to block non-specific staining, then on drops of mouse anti-GFP, washed and floated on drops of goat ant-mouse Ig conjugated to 10 nm gold particles. After final washing, sections were stained with uranyl acetate and examined in a Jeol 1200EX electron microscope.

### Immunofluorescence assay

IFAs were performed on air dried ookinete slides. Ookinete cultures were produced as described above. Cells were fixed in 4% paraformaldehyde in MTSB buffer (Guttery et al., 2012) for 10 min and permeabilised with 0.2% Triton-X-100. Primary antibodies used were mouse monoclonal anti-α-tubulin (1:500, Sigma), mouse monoclonal anti-GFP (1:650, Invitrogen), rabbit anti-GFP (1:250, Invitrogen) and rabbit anti-GAP45 (1:250 (Green et al., 2008)). Secondary antibodies were Alexa Fluor 488 goat anti-rabbit, Alexa Fluor 568 goat anti-rabbit, Alexa Fluor 488 goat anti-mouse and Alexa Fluor 568 goat anti-mouse (all 1:1,000, Invitrogen). Cells were counterstained with DAPI (Invitrogen).

### Liver stage cultures

For analysis of liver stages, HepG2 cells cultured in DMEM medium supplemented with FCS and antibiotics were infected with sporozoites and incubated at 37°C for 5 to 68 h. Cells were fixed with 4% paraformadehyde and permeabilized with Triton X-100 before analysis by immunofluorescence using anti-HSP70 antibodies (Tsuji et al., 1994) and the nuclear stain Hoechst 33342 (Invitrogen). Liver merozoites/merosomes were recovered from culture supernatants at 68 h post-infection and stained with Hoechst 33342 before examination by fluorescence microscopy. Images were acquired on a DMI inverted fluorescence microscope (Leica) equipped with an AxioCam MRC5 camera (Zeiss).

### qRT-PCR analysis

Parasites were purified from blood (see above) and the RNA was isolated using the Absolutely RNA purification kit (Stratagene). cDNA was synthesised using an RNA-to-cDNA kit (Applied Biosystems). Gene expression was quantified from 250 ng of total RNA. qPCR reactions consisted of 2 µl of cDNA, 5 µl SYBR green fast master mix (Applied Biosystems), 0.5 µl (250 nM) each of the forward and reverse primers, and 2 µl DEPC-treated water. All of the primers were designed with primer3 (Primer-blast, NCBI), and amplified a region 70–200 bp. Analysis was conducted using an Applied Biosystems 7500 fast machine with the following cycling conditions: 95°C for 20 sec followed by 40 cycles of 95°C for 3 sec; 60°C for 30 sec. Three biological replicates were performed for each assayed gene. Wild-type expression was determined using the Pfaffl method (Pfaffl, 2001). Relative quantification in the mutant line was compared to wild-type expression using the ΔΔC_t_ method. Both methods used *hsp70* (PBANKA_081890) (forward, 5′-GTATTATTAATGAACCCACCGCT-3′; reverse, 5′-GAAACATCAAATGTACCACCTCC-3′) and *arginyl-tRNA synthetase* (PBANKA_143420) (forward, 5′-TTGATTCATGTTGGATTTGGCT-3′; reverse, 5′-ATCCTTCTTTGCCCTTTCAG-3′) as reference genes. The *isp1* primers were: forward, 5′-GCCACCAAAAGGTACGAATG-3′; reverse, 5′-GCCAAACAACAATTGCCACT-3′. The *isp3* primers were: forward, 5′-AGCTTGTGCTGCATTAACGA-3′, reverse, 5′-TTGAATTTCATTTCCATCAGGA-3′.

### Statistical analysis

All statistical analyses were performed using GraphPad Prism 5 (GraphPad Software). For qRT-PCR, an unpaired *t*-test was conducted to show significant differences between wild-type and mutant strains.

## Supplementary Material

Supplementary Material
