# Supplementary material for: Unique apicomplexan IMC sub-compartment proteins are early markers for apical polarity in the malaria parasite
Source: Biol Open. 2013 Sep 16;2(11):1160–70. doi: 10.1242/bio.20136163 (PMC3828762; doi:10.1242/bio.20136163)
Supplement: Supplementary Material [file supp_2_11_1160_v2_index.html]

Unique apicomplexan IMC sub-compartment proteins are early markers for apical polarity in the malaria parasite — Supplementary Material 

# Unique apicomplexan IMC sub-compartment proteins are early markers for apical polarity in the malaria parasite

## bio.20136163 Supplementary Material

**Files in this Data Supplement:**

- Supplementary Material - Benoit Poulin et al. doi: 10.1242/bio.20136163
