# Supplementary material for: Unique apicomplexan IMC sub-compartment proteins are early markers for apical polarity in the malaria parasite
Source: Biol Open. 2013 Sep 16;2(11):1160–70. doi: 10.1242/bio.20136163 (PMC3828762; doi:10.1242/bio.20136163)
Supplement: Supplementary Material [file supp_bio.20136163_bio.20136163-s1.pdf]

Supplementary Material

Benoit Poulin et al. doi: 10.1242/bio.20136163

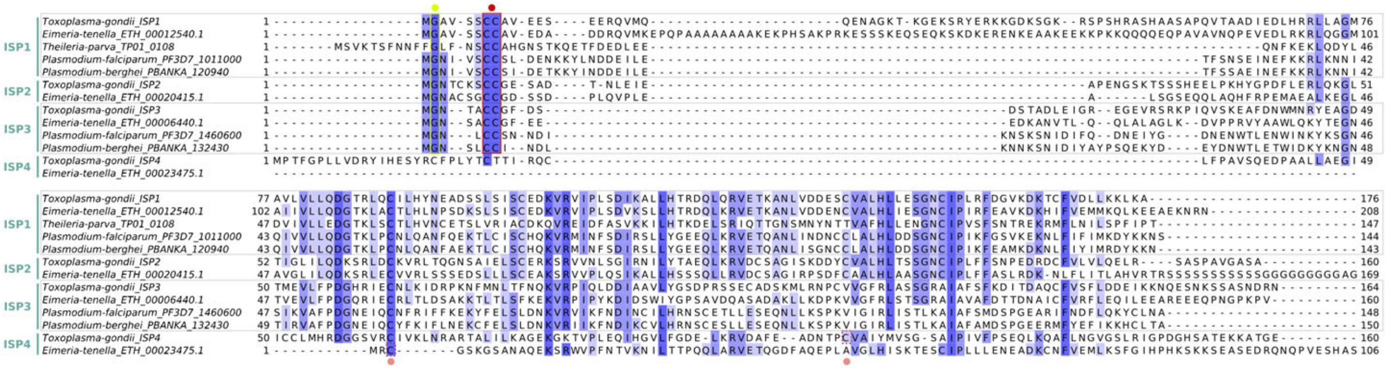

Fig. S1. Phylogenetic analysis of ISP proteins in *Apicomplexa*. Residues are coloured according to degree of conservation. The positions of predicted sites for myristoylation (yellow) and palmitoylation (red) are also shown. ISP4 sequences lack strong predictions for palmitoylation, but potential other sites for S-palmitoylation are indicated (pink dot). For convenience, the C-terminal extension of *Eimeria* ISP2 is not included.

Biology Open

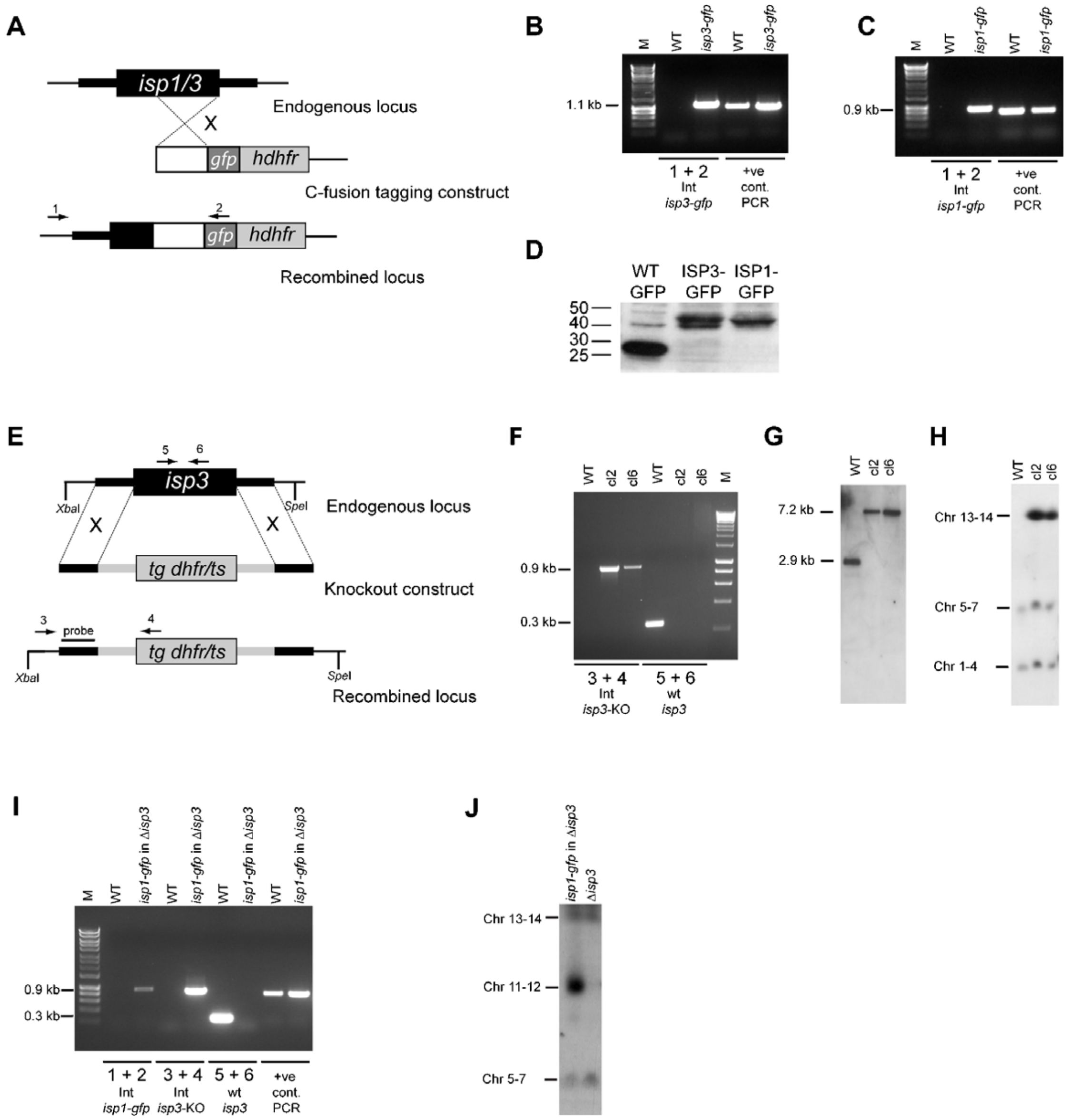

Fig. S2. Generation of transgenic *isp* lines. A) Schematic representation of the gene targeting strategy employed for tagging the endogenous *isp1* and *isp3* loci with *gfp* via single homologous recombination. The C-fusion tag construct contains an insert (white box) homologous to the 3′ end of the *isp1* or *isp3* ORF fused to *gfp*. A human dihydrofolate reductase selectable marker (*hdhfr*) allows for selection of transgenic parasites. Arrows 1 and 2 indicate primers used for diagnostic PCR. B) Diagnostic integration PCR for *isp3-gfp* parasites showing the expected 1.1 kb integration band (primers 1 and 2), thus confirming successful integration of the tagging construct. A second set of primers was used as positive control (+ve cont.) to amplify an unrelated locus to confirm presence of DNA in the PCR mix. Wild type (WT) gDNA was used as control. C) Diagnostic integration PCR for *isp1-gfp* parasites showing the expected 0.9 kb integration band (primers 1 and 2), thus confirming successful integration of the tagging construct. A second set of primers was used as positive control (+ve cont.) to amplify an unrelated 1 kb locus to confirm presence of DNA. Wild type (WT) gDNA was used as control. D) Western blot analysis using an anti-GFP (Invitrogen) antibody against protein extracted from blood infected with WT *P. berghei* ANKA 507 clone 1 constitutively expressing GFP (WT-GFP) and transgenic (ISP1-GFP and ISP3-GFP) parasites showing bands of expected size of 29 kDa for wild-type-GFP, 46.5 for ISP3-GFP and 45.4 kDa for ISP1-GFP. E) Schematic representation of the endogenous *isp3* locus, the knockout construct and the recombined *isp3* locus following double cross-over recombination. The knockout construct contains a *T. gondii* dihydrofolate reductase/thymidylate synthase (*tg dhfr/ts*) cassette with a Pbdhfr 3′ UTR for selection of transgenic parasites with pyrimethamine. Arrows 3, 4, 5 and 6 indicate binding sites for primers used in integration PCR and knockout PCR. *Xba*I and *Spe*I restriction sites and probe binding sites used for Southern blot analysis are shown. F) Genotypic analysis of *Δisp3* parasites by integration PCR and knockout PCR. Presence of a 0.9 kb band using integration specific primers 3 and 4 (Int *isp3*-KO) on gDNA of mutants (cl2 and cl6) confirms correct integration of the targeting construct. Absence of the 0.3 bp wild type specific band amplified by primers 5 and 6 (wt *isp3*) in cl2 and cl6 shows loss of the endogenous *isp3* gene. Wild type (WT) gDNA was used as control. G) Genotypic analysis of *Δisp3* parasites by Southern blot. gDNA of wild type (WT) parasites, *Δisp3* mutants cl2 and cl6 was probed following *Xba*I and *Spe*I digestion. The probe homologous to the *isp3* 5′ UTR recognizes a 2.9 kb fragment for the endogenous locus and a 7.2 kb fragment for the recombined locus. H) Pulse-field gel electrophoresis blot (PFGE) hybridized with a *Pbdhfr* 3′UTR probe. The probe hybridizes to the endogenous *dhfr* locus on chromosome 7 and the *gfp* cassette integrated in the 230p locus of the parental line PbANKA 507 clone 1 as well as the disrupted *isp3* locus on chromosome 13.
I) Diagnostic PCR for *isp1-gfp* tagged parasites in *Δisp3* genetic background. Successful integration of the *isp1* tagging construct is confirmed by a 0.9 kb band amplified with primers 1 and 2 (Int *isp1-gfp*). Integration of the *isp3* knockout construct is confirmed by a 0.9 kb band amplified with primers 3 and 4 (Int *isp3*-KO), whereas the loss of the endogenous *isp3* is confirmed by the absence of a 0.3 kb band amplified by primers 5 and 6 (wt *isp3*). A fourth set of primers was used as positive control (+ve cont.) to amplify an unrelated locus to confirm presence of DNA. Wild type (WT) gDNA was used as control. J) PFGE for *isp1-gfp* tagged parasites in *Δisp3* genetic background hybridized with the same *Pbdhfr* 3′UTR probe as in H). The probe hybridizes to the disrupted *isp3* locus on chromosome 13 and to the *isp1-gfp* on chromosome 12.

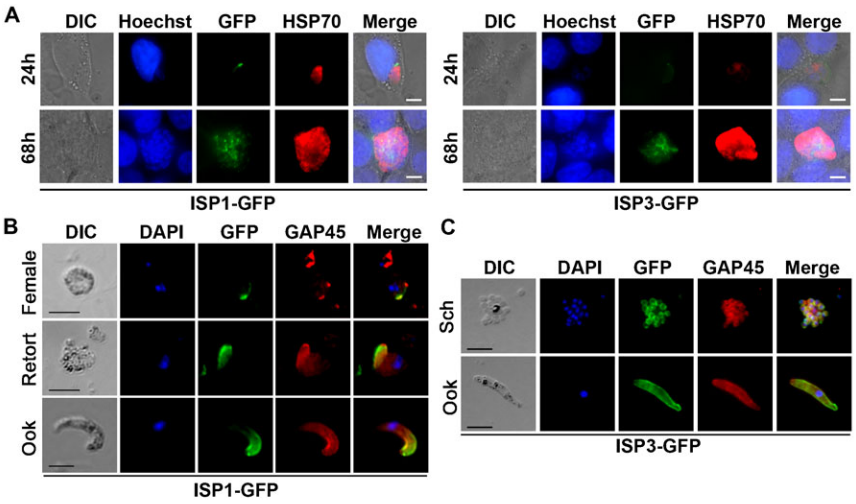

Fig. S3. Liver stages and indirect immunofluorescence with GAP45 antibodies. A) Expression of ISP1-GFP and ISP3-GFP in hepatocytes *in vitro* at 24 h and 68 h (Scale bar = 5 µm). B) Indirect immunofluorescence of ISP1-GFP with respect to the IMC marker GAP45, at gametocyte and ookinete stages (Scale bar = 5 µm). Co-localisation with GAP45 is observed at the apical end of the parasite. C) Co-staining of ISP3-GFP with GAP45, at schizont and ookinete stage parasites (Scale bar = 5 µm). In schizonts, the GAP45 staining overlaps with the ISP3-GFP at the membrane of the individual merozoites. At ookinete stage, ISP3-GFP co-localises with the IMC marker at the apical end.

Biology Open
